# Recent Advances in Studying Age-Associated Lipids Alterations and Dietary Interventions in Mammals

**DOI:** 10.3389/fragi.2021.773795

**Published:** 2021-11-19

**Authors:** Benedikt Gille, Christina E. Galuska, Beate Fuchs, Shahaf Peleg

**Affiliations:** ^1^ Research Group Epigenetics, Metabolism and Longevity, Research Institute for Farm Animal Biology (FBN), Dummerstorf, Germany; ^2^ Core Facility Metabolomics, Research Institute for Farm Animal Biology (FBN), Dummerstorf, Germany; ^3^ Institute of Neuroregeneration and Neurorehabilitation, Qingdao University, Qingdao, China

**Keywords:** aging, lipid, healthy life span, metabolism, dietary intervention

## Abstract

Lipids are involved in a broad spectrum of canonical biological functions, from energy supply and storage by triacylglycerols to membrane formation by sphingolipids, phospholipids and glycolipids. Because of this wide range of functions, there is an overlap between age-associated processes and lipid pathways. Lipidome analysis revealed age-related changes in the lipid composition of various tissues in mice and humans, which were also influenced by diet and gender. Some changes in the lipid profile can be linked to the onset of age-related neurodegenerative diseases like Alzheimer’s disease. Furthermore, the excessive accumulation of lipid storage organelles, lipid droplets, has significant implications for the development of inflammaging and non-communicable age-related diseases. Dietary interventions such as caloric restriction, time-restrictive eating, and lipid supplementation have been shown to improve pertinent health metrics or even extend life span and thus modulate aging processes.

## Introduction

Lipids are an elementary component of all organisms and are involved in a variety of organismal processes. The development of novel high throughput and sensitive detection methods in combination with genetically modified model organisms led to a wide range of discoveries in the field of lipid research in recent years (Vinayavekhin et al., 2010; Pamplona et al., 2019). Due to the high variability of combinations of fatty acids, head groups and other compounds, a high theoretical number of about 180,000 different lipid species could be potentially involved in biochemical processes (Brügger, 2014). Importantly, previous studies suggested an overlap between lipid-connected processes and pathways associated to aging (de Diego et al., 2019).

During aging, lipid metabolism and cell membrane composition of different tissues undergo substantial measurable changes that impact the functionality of relevant organs like the brain or heart in humans (Almeida et al., 2021). Such age-associated alterations are determined by a variety of factors like genetic background and gender (Nam et al., 2017; Wong et al., 2020). Indeed, these effects contribute to the development of species-specific aging phenotypes, often associated with the accumulation of molecular and cellular damage and a deterioration of functions, rendering the individual more vulnerable to age related diseases and increasing the probability of death (Lemoine, 2020). The investigation of the lipidome in progeroid models and longitudinal studies revealed systematic changes in metabolism and membrane lipid profile during aging processes (Almeida et al., 2021). Understanding the modes of action of lipids in age-associated alterations can help to explain established interventions and suggests novel approaches for treatments to extend health- and life span.

Since diet is an important factor influencing lipid metabolism during aging, dietary interventions such as caloric restriction (CR) or time-restrictive eating may be applied as health- and life span extending therapies (de Diego et al., 2019). For example, intermittent fasting (IF), which describes a daily cycle of extended fasting with a shortened time frame of food intake, gained considerable attention recently (Meng et al., 2020). After providing a general classification of lipids involved in biological processes, this review will summarize recent findings in age-dependent changes in lipid metabolism and advances in understanding the role of lipids in dietary longevity interventions.

## Classification and Roles of Lipids in Animal Tissue and Cells

**FIGURE 1:**
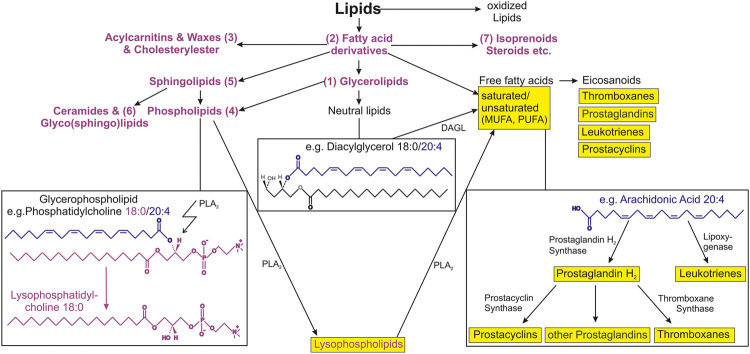
Schematic overview of lipid subclasses and connections to selected lipid metabolic pathways. Lipids are very diverse and complex biological compounds. The arrows illustrate similarities and connection between lipid categories, main classes, subclasses and substructures. Seven categories are shown that have been relevant in aging-associated studies. Examples for phospholipids and neutral lipids (as a subclass of glycerophospholipids) are shown in the left and middle box, respectively. The right box shows the simplified arachidonic cascade as example for the eicosanoid production from free fatty acids. Important lipid mediators that are known to be involved in inflammatory processes are shown in yellow. Abbreviations: PLA_2_, phospholipase A_2_; MUFA, monounsaturated fatty acid; PUFA, polyunsaturated fatty acid; DAGL, diacylglycerol lipase.

Lipids are mostly hydrophobic (water-insoluble) biomolecules due to the long carbon residues that most of the lipids possess. Based on their low polarity, lipids are soluble in hydrophobic (lipophilic) solvents such as hexane. In living organisms, lipids are predominantly constituents of biological membranes, serve as signaling molecules or energy storage and source and are an important and essential nutrient (Berg et al., 2002). Lipids can be classified into mainly seven subclasses: triacylglycerols (oils and fats) (1), fatty acids (and derivatives) (2), waxes (3), phospholipids (4), sphingolipids (5), glycolipids (6) and isoprenoids (carotenoids and steroids) (7). An overview of all subclasses is illustrated in Figure 1.

The bulk of the nutritional lipids are the neutral glycerolipids such as triacylglycerols (1) with more than 90%. They provide more energy (39 kJ per 1 g fat) than other nutrients such as sugars or proteins (17 kJ per 1 g sugar or protein). Therefore, triacylglycerols are the most important energy storage of the body and insulate the body and organs against cold or injury. While often, the term fat is used as synonym for lipids, only one of the subgroups namely triacylglycerol is considered as fat. Chemically, fats and fatty oils are triple esters of the glycerol (esters follow the schema R1-CO-O-R^2^) and are therefore called triacylglycerols. Triacylglycerols are called “simple” if they have identical fatty acyl side chains, or “mixed” if they are different. Furthermore, they can be divided into liquid (oil) when the portion of unsaturated fatty acyl side chains are higher or hard (fat) when the portion is lower. Unsaturated fatty acids often exhibit *cis* double bonds that hinder crystallization and lower the melting point: the more double bonds, the lower the melting point. Saponified triacylglycerols are cleaved into glycerol and the corresponding fatty acids (Lichtenstein, 2013).

Fatty acids (2) are mainly unbranched mono carbonic acids–a (long) carbon chain with a carboxyl group at the end and are divided into saturated fatty acids without double bonds and unsaturated fatty acids with one or two or more (in nature mainly not conjugated) double bonds. They serve in the *ß*-oxidation process as fuel (Schulz, 2013). Metabolically relevant unsaturated fatty acids are for example oleic acid (18:1) and arachidonic acid (20:4). The synthesis of unsaturated fatty acids in higher animals is limited and must be secured for the so-called “essential fatty acids” through food intake. Essential *ω*-3 fatty acids are linolenic (18:3), eicosapentaenoic (20:5), docosahexaenoic acid (22:6), *ω*-6 fatty acids linoleic (18:2) and arachidonic acid (20:4), the latter one is precursor for eicosanoids, which are important tissue hormones and mediators in animal bodies (Park and Chalfant, 2018).

Waxes (3) are monoesters of fatty acids with long saturated alkyl residues on both the acid and the alcoholic part and less oily and more rigid and porous than triacylglycerols (Mortimer and Müller, 2003).

Phospholipids (4), sphingolipids and glycolipids belong to the membrane-forming lipids, and in contrast to triacylglycerols, they contain both hydrophilic and hydrophobic groups and are therefore amphiphilic. They form micelles or double lipid layers in polar solvents such as water, which is the basis for all biomembranes for isolating cells of their environment and the basic requirement of all living organisms. All glycerophospholipids are made of a glycerol backbone, where the first and second hydroxyl group is esterified with two variable fatty acyl residues representing the hydrophobic part and the remaining hydroxyl position with phosphoric acid (hydrophilic). The resultant lipid subclass is phosphatidic acid from that other glycerophospholipids can be formed *via* ester bond with various alcohols (hydrophilic head groups) such as choline or ethanolamine resulting in phosphatidylcholine or phosphatidylethanolamine, respectively. The two lipid subclasses represent the most abundant glycerophospholipid subgroups in bio membranes; further head groups are serine (phosphatidylserine), inositol (phosphatidylinositol) and inositol with up to three phosphate groups at the inositol ring (polyphosphoinositide). The last plays a major role in signal transduction in cells.

Beside phospholipids with glycerol backbone, another significant phospholipid without glycerol is sphingomyelin that belongs also to the sphingolipids (5) that are composed of a fatty acid and sphingosine. Subgroups of sphingolipids are ceramides, sphingomyelins and glycolipids (6) that are important for the nerve tissue in signal transduction between cells. Glycolipids are phosphate free sphingosine-containing lipids where a carbohydrate group is bound to the 1-hydroxy group of the sphingosine (Jing et al., 2015). The bulk of phospholipids in nature is esterified with a saturated fatty acyl chain in the first glycerol position, whereas in the second with an often moderately unsaturated (e.g. 18:1) or even highly unsaturated (e.g. 20:4). Beside diacyl phospholipids there are also alkyl-acyl and alkenyl-acyl glycerophospholipids and such compounds are called ether lipids or plasmalogens (Lessig and Fuchs, 2009).

Phospholipids are transformed into lysophospholipids by the cleavage mediated by phospholipases and the compounds derived of the cleavage of sphingomyelins are sphingosine-1-phosphate and ceramide that possess important cellular functions (Billich and Baumruker, 2008). Phospholipases C and D lead to the generation of diacylglycerols and phosphatidic acid that are important signaling molecules (Wymann and Schneiter, 2008). The enzymatic (phospholipase A_2_) released (usually unsaturated) fatty acyl chains exhibit further great biological importance: unsaturated fatty acids like arachidonic acid are easily oxidizable and their metabolic pathway lead to eicosanoids, prostaglandins, thromboxanes or leukotrienes that have significant physiological impact e.g. as hormones (Arab and Akbar, 2002).

Steroids and carotenoids belong to the lipid group of isoprenoids (7). In nature, occurring steroids belong to the triterpenoid (composed of 30 carbon atoms) derivatives, whereas carotenoids belong to the tetraterpenoid derivatives (composed of 40 carbon atoms). The basic structure of all steroids is composed of four carbon rings, three hexagonal and one pentagonal ring. The most well-known steroid is cholesterol that is an essential component of all biological membranes, except of the inner membrane of mitochondria, and belongs therefore to the membrane-forming lipids. Further steroids are the sex hormones such as the female sex hormones progesterone and estrogen, as well as the male androgens testosterone and androsterone. Other examples are the sterines ergosterole, phytosterine and vitamin D. The latter one is important for the regulation of the calcium and phosphorus concentration in the blood and bone stability.

Of note, the yellow to reddish pigments in plants are carotenoids and exclusively synthesized by them. The most well-known pigment is *ß*-carotene also known as provitamin A that is converted in animals into vitamin A and is important for the viewing process as well as for the skin and mucous membranes (Handa et al., 2014).

## Lipids Alterations During Aging

Age-related and tissue-specific changes in lipid composition can contribute to the aging process. The quantification of these alterations in the lipidome in healthy aging organisms revealed a general systematic trend in lipid profile changes (Almeida et al., 2021; Chung, 2021). Of note, the lipidome composition itself and age-related changes can be impacted by different factors like diet (Nam et al., 2017; Surma et al., 2021) and genetic background (Wong et al., 2020; McGurk et al., 2021). Importantly, gender emerged as a major determinator of the plasma lipidome. For example, in women, the plasma lipidome is subjected to stronger changes than in men during aging (Jové et al., 2016; Audano et al., 2018). Aged women display a higher increase in plasma triglycerides and phospholipids compared to aged men, partially due to severely decreased estrogen levels during menopause (Kolovou and Bilianou, 2008; Slade et al., 2021). The more pronounced changes in female brain membrane lipidome could potentially underlie the higher prevalence of Alzheimer’s disease in postmenopausal women (Díaz et al., 2018).

In fact, the aging mammalian brain membranes in general are subjected to moderate changes, which nonetheless can have extensive impact on cognitive health. Essentially, the proportions of polyunsaturated fatty acids (PUFAs), short-chained sphingolipids, cholesterol and phospholipids decrease in aging mouse and human brains and human cerebrospinal fluid, while the concentration of long-chained sphingolipids and monounsaturated fatty acids (MUFAs) tend to increase (Tu et al., 2017; Pamplona et al., 2019; Hwangbo et al., 2021; Jové et al., 2021). These cerebral lipidome alterations presumably contribute to age-related neuronal deterioration by causing mitochondrial dysfunction, increasing oxidative stress and altering properties of neuronal membranes. For example, lower levels of the phospholipid cardiolipin in the mitochondrial membranes of normally aging brains is associated with a reduction of electron transport chain activity, an observation also made in brains of patients with Alzheimer’s disease (Kao et al., 2020). Additionally, the decrease in PUFAs and different phospholipids with age reduces fluidity of neuronal membranes, which directly reduces diffusion of membrane proteins, alters protein-protein interaction, and thus changes neuronal signaling with negative implications on cognitive function (Céspedes et al., 2021; Das, 2021). In lipid rafts, changes in the lipidome may have even stronger effects. Lipid rafts are microdomains in membranes with concentrated protein complexes for signal regulation and transduction cascade. Age-correlated and progeroid lipidome alterations, especially the reduction in cholesterol, impaired neuronal physiology and function in mice and is strongly associated with Alzheimer’s diseases and Parkinson’s disease (Mesa-Herrera et al., 2019; Poljak et al., 2020; Jové et al., 2021).

The underlying mechanisms behind the cerebral membrane composition changes still need to be clarified. In the case of cholesterol, remodeling in homeostasis pathways and synthesis are responsible for the depletion that is observed in cell membranes of some brain regions. The downregulation of the transporter ApoE and synthesis and the upregulation of the cholesterol-removing enzyme CYP46 in aged humans contributed to lower local cholesterol concentration in the brain (Martin et al., 2010; Jové et al., 2021). PUFAs originate either from synthesis in the liver or from diet, and in both cases must pass the blood-brain barrier (BBB) to be incorporated in cerebral cell membranes. While the plasma PUFA level increases in older humans, brain membranes contain less with age (Chappus-McCendie et al., 2019), indicating potential alterations in the transport across the BBB. Indeed, the BBB undergoes morphological and functional changes during aging that impair lipid transportation pathways, but the exact causes remain to be resolved (Pifferi et al., 2021).

Microglia, the immune cells of the central nervous system, play an important role in the maintenance of the brain and their dysfunction is causally linked to the onset of neurodegeneration (Hickman et al., 2018). Microglia have been recently shown to accumulate lipid droplets (LD) in mouse and human brains (Marschallinger et al., 2020). While the canonical function of these organelles is the storage of lipids like triacylglycerols and cholesteryl esters for metabolism and membrane formation (Welte and Gould, 2017), the excessive accumulation in microglia led to their functional decline and a pro-inflammatory cytokine profile. These LD accumulating microglia and other cerebral cells could contribute to the deterioration of the central nervous system and the onset of neurodegenerative diseases (Farmer et al., 2020; Marschallinger et al., 2020). Overall, the accumulation of LD in tissues like kidney, liver, muscles and immune cells like monocytes is associated with an impaired fatty acid oxidation through the downregulation of the peroxisome proliferator-activated receptor PPAR-*α* (Marschallinger et al., 2020; Chung, 2021; Wang et al., 2021). In the case of monocytes, the reduced expression of PPAR-*α*, accompanied by LD accumulation, leads to a pro-inflammatory polarization of these immune cells. This could contribute to inflammaging, thus increasing the risk of age-related diseases (Wang et al., 2021).

Cardiovascular diseases, of which coronary artery disease is the most common and leading cause of death worldwide (WHO, 2020), are caused among other factors, by unfavorable lipid profiles and the accumulation of lipids in the tissue (Britton and Fox, 2011). In particular, low high-density lipoprotein cholesterol, high low-density lipoprotein cholesterol and high triacylglycerol levels in blood were associated with higher incidence of cardiac diseases (Kaneko et al., 2021). In humans, myocytes are particularly prone to ectopic LD accumulation (Pieńkowska et al., 2019). The accumulation of ectopic LD in human myocytes leads to a modification of lipid metabolism and consequently contributes to a reduced insulin sensitivity, a hallmark for type 2 diabetes, even in non-obese subjects (Gemmink et al., 2017; Ferrara et al., 2019). On the other hand, moderate lipid accumulation, as in the epicardial adipose tissue (EAT), is beneficial for coronary artery protection and energy supply. However, if the lipid supply exceeds the storage and oxidative capacity of the EAT, lipotoxic molecules induce apoptosis which is associated with atrial fibrillation (Ferrara et al., 2019). Recently, the histone deacetylase 6 (Hdac6) was identified as a possible mediator of lipid droplet formation in flies. Together with p62, an autophagy receptor protein, Hdac6 probably regulates selective autophagy of LD in oenocytes (Yan et al., 2017; Yan et al., 2019).

## Therapeutic Interventions Targeting Lipids to Improve Healthy Life Span

The most prominent dietary interventions for health and life span extension in most model organisms are CR and time-restrictive eating. Such interventions show positive effects in animal models on autophagy, systemic inflammation and nutrient sensing (Di Francesco et al., 2018; Chung et al., 2020). Another approach is the supplementation or avoidance of certain nutrients to counteract age-related changes (Bruins et al., 2019; Johnson and Stolzing, 2019).

CR and intermittent fasting (IF) are known to cause various alterations in systemic processes that may have an effect on aging and health span, while the specific modes of action are still being studied (Chung et al., 2020). These effects of CR and IF have shown to have beneficial impact in many model organisms across different taxa (Hwangbo et al., 2020). However, CR and IF are by no means universal and such interventions could have neutral or even negative impact on many strains of mice (Liao et al., 2010). Nonetheless, lipid metabolism and homeostasis are a possible link between the interventions and a deceleration of aging processes. The feeding of 60% calorie restricted diet or time-restrictive feeding for 24 h on three non-consecutive days per week reduced the proportion of total adipose tissue and promoted the browning of white adipose tissue in mice, which is associated with a healthier phenotype and improved insulin sensitivity (Fabbiano et al., 2016; Liu et al., 2019). In contrast, data in human subcutaneous adipose tissue did not show such an effect after an 8-weeks low-calorie diet of 800 kcal/day (Barquissau et al., 2018). In line with this, a recent study in healthy mice showed that a 30% calorie reduced diet did not cause metabolic changes and life-extension, but rather the 30% calorie reduced diet in combination with fasting in daily cycles (Pak et al., 2021). Still, these interventions have the potential to reduce inflammaging by diminishing the amount of pro-inflammatory adipokine-releasing white adipose tissue (Zamboni et al., 2021). Additionally, recent meta-analyses showed the lipid-profile altering properties of IF and CR in humans. For example, Meng et al. and others showed that different types of IF and CR can significantly improved the serum lipid-profile by reducing triacylglycerol, total cholesterol and low-density lipoprotein cholesterol concentrations, which are associated with age-related diseases (Wadhera et al., 2016; Almeida et al., 2021). Of note, high-density lipoprotein cholesterol concentrations in human serum were not affected by different versions of these interventions (Meng et al., 2020).

Different types of IF and CR can substantially decrease the risk factor to suffer from diabetes, inflammation and impaired balance and movement control (Zubrzycki et al., 2018; Becker et al., 2021). The underlying mechanisms of these observations are thought to be similar to the causes of LD accumulation. An increased expression of PPAR-*α* during IF upregulates the production of enzymes involved in *ß*-oxidation of fatty acids, thus lowering free fatty-acids (Meng et al., 2020). Furthermore, higher expression of hepatic PPAR-*α*, induced by intermittent fasting (20 h feeding +4 h fasting, 4 h feeding +20 h fasting and 24 h feeding +24 h fasting), decreased systemic inflammation by reducing the number of circulating monocytes in mice (Jordan et al., 2019). A recent study in mice showed that even modest and relatively late dietary intervention (*ad libitum* of 16.4% calorie reduced feed, starting at 12 weeks of age) in Titan obese mice is sufficient to reduce lipid content and improve life span (Müller-Eigner et al., 2021).

Likewise, the supplementation of specific lipid classes in addition to a balanced diet can prevent the development of age-related diseases and influence life span. Accordingly, a recent study in *Drosophila melanogaster* suggested that the shortened life span of female flies with a diet of high protein: carbohydrate ratio is improved by diet supplement of cholesterol (Zanco et al., 2021). A recent review regarding the impact of certain macro- and micronutrients on age-related noncommunicable diseases by Bruins et al. (2019) discussed the vital role of many lipid classes in basic nutrition. Epidemiological studies emphasized excess intake of saturated fatty acids and increased blood low-density lipoprotein cholesterol levels as a major factor for the development of cardiovascular diseases, while the higher consumption of long-chain PUFAs and MUFAs in a population is associated with a lower prevalence of type 2 diabetes, hypertension and cardiovascular diseases (Bruins et al., 2019; Clifton, 2019). The modulation of the pro-inflammatory profile of adipocytes to reduce the secretion of the inflammation-regulating adipokines leptin and adiponectin is a prominent target by dietary interventions. In a recent review of randomized, controlled trials testing the supplementation of different PUFAs on the circulating levels of these adipokines Rausch et al. discussed the considerable influence of dose and duration of intake on the outcome of these studies. Despite appreciable variations in the results of the studies included in the review, the supplementation of different PUFAs appears to be a reasonable measure to reduce adipocyte-induced inflammaging (Rausch et al., 2021).

## Conclusion

Recent research emphasized the key role of lipids in biogerontology. Since lipids are involved in a variety of biochemical processes, it is not surprising that lipids provide a link between homeostasis and age-related phenotypes. Current studies continue expanding our knowledge and revealing novel connections between lipids and aging. Recent data supports the notion that age-related lipidome changes and the accumulation of LD in microglia in the brain can have substantial influence on the onset of incurable neurodegenerative diseases by remodeling cerebral cell membranes and increasing inflammation. Additionally, LD accumulation in myocytes is associated with a reduced insulin sensitivity in humans, linking lipid metabolism to type 2 diabetes.

Lipid-related interventions, which include caloric- or time-restricted eating or the supplementation of certain lipids, will continue to be investigated in the context of health. Experimental and epidemiological studies showed that these interventions could increase health- and life span by targeting lipid homeostasis and metabolism. Indeed, the effectiveness of such interventions is already well studied in mammalian animal models. One exciting notion is to characterize individual genomes, identify potential links between the personal genome and its potential impact on lipid metabolism, and based on that, generate personalized nutrition that can sustain a healthier diet and lipid profile throughout time (dnaforme.com). Currently, further clinical and longitudinal studies are required in humans to clarify the preventative or therapeutic properties of specific diets or lipid supplementations in extending human health and life span.

## References

[B1] Almeida I. Magalhães S. Nunes A. (2021). Lipids: Biomarkers of Healthy Aging. Biogerontology 22, 273–295. 10.1007/s10522-021-09921-2 33837874

[B2] Arab L. Akbar J. (2002). Biomarkers and the Measurement of Fatty Acids. Public Health Nutr. 5, 865–871. 10.1079/PHN2002391 12638594

[B3] Audano M. Maldini M. De Fabiani E. Mitro N. Caruso D. (2018). Gender-related Metabolomics and Lipidomics: From Experimental Animal Models to Clinical Evidence. J. Proteomics 178, 82–91. 10.1016/j.jprot.2017.11.001 29122727

[B4] Barquissau V. Léger B. Beuzelin D. Martins F. Amri E.-Z. Pisani D. F. (2018). Caloric Restriction and Diet-Induced Weight Loss Do Not Induce Browning of Human Subcutaneous White Adipose Tissue in Women and Men with Obesity. Cel Rep. 22, 1079–1089. 10.1016/j.celrep.2017.12.102 29386128

[B5] Becker A. Gaballa D. Roslin M. Gianos E. Kane J. (2021). Novel Nutritional and Dietary Approaches to Weight Loss for the Prevention of Cardiovascular Disease: Ketogenic Diet, Intermittent Fasting, and Bariatric Surgery. Curr. Cardiol. Rep. 23, 85. 10.1007/s11886-021-01515-1 34081228

[B6] Berg J. M. Tymoczko J. L. Stryer L. (2002). Biochemistry. New York, NY: W. H. Freeman.

[B7] Billich A. Baumruker T. (2008). Sphingolipid Metabolizing Enzymes as Novel Therapeutic Targets. Subcell Biochem. 49, 487–522. 10.1007/978-1-4020-8831-5_19 18751924

[B8] Britton K. A. Fox C. S. (2011). Ectopic Fat Depots and Cardiovascular Disease. Circulation 124, e837–e841. 10.1161/CIRCULATIONAHA.111.077602 22156000

[B9] Brügger B. (2014). Lipidomics: Analysis of the Lipid Composition of Cells and Subcellular Organelles by Electrospray Ionization Mass Spectrometry. Annu. Rev. Biochem. 83, 79–98. 10.1146/annurev-biochem-060713-035324 24606142

[B10] Bruins M. J. van Dael P. Eggersdorfer M. (2019). The Role of Nutrients in Reducing the Risk for Noncommunicable Diseases during Aging. Nutrients 11, 85. 10.3390/nu11010085 PMC6356205 30621135

[B11] Céspedes P. F. Beckers D. Dustin M. L. Sezgin E. (2021). Model Membrane Systems to Reconstitute Immune Cell Signaling. Febs J. 288, 1070–1090. 10.1111/febs.15488 32681663

[B12] Chappus-McCendie H. Chevalier L. Roberge C. Plourde M. (2019). Omega-3 PUFA Metabolism and Brain Modifications during Aging. Prog. Neuro-Psychopharmacology Biol. Psychiatry 94, 109662. 10.1016/j.pnpbp.2019.109662 31152862

[B13] Chung H. Y. Kim D. H. Bang E. Yu B. P. (2020). Impacts of Calorie Restriction and Intermittent Fasting on Health and Diseases: Current Trends. Nutrients 12, 2948. 10.3390/nu12102948 PMC7599444 32992924

[B14] Chung K. W. (2021). Advances in Understanding of the Role of Lipid Metabolism in Aging. Cells 10, 880. 10.3390/cells10040880 33924316 PMC8068994

[B15] Clifton P. (2019). Metabolic Syndrome-Role of Dietary Fat Type and Quantity. Nutrients 11, 1438. 10.3390/nu11071438 PMC6683280 31247933

[B16] Das U. N. (2021). "Cell Membrane Theory of Senescence" and the Role of Bioactive Lipids in Aging, and Aging Associated Diseases and Their Therapeutic Implications. Biomolecules 11, 241. 10.3390/biom11020241 33567774 PMC7914625

[B17] de Diego I. Peleg S. Fuchs B. (2019). The Role of Lipids in Aging-Related Metabolic Changes. Chem. Phys. Lipids 222, 59–69. 10.1016/j.chemphyslip.2019.05.005 31152691

[B18] Di Francesco A. Di Germanio C. Bernier M. de Cabo R. (2018). A Time to Fast. Science 362, 770–775. 10.1126/science.aau2095 30442801 PMC8504313

[B19] Díaz M. Fabelo N. Ferrer I. Marín R. (2018). "Lipid Raft Aging" in the Human Frontal Cortex during Nonpathological Aging: Gender Influences and Potential Implications in Alzheimer's Disease. Neurobiol. Aging 67, 42–52. 10.1016/j.neurobiolaging.2018.02.022 29627763

[B20] Fabbiano S. Suárez-Zamorano N. Rigo D. Veyrat-Durebex C. Stevanovic Dokic A. Colin D. J. (2016). Caloric Restriction Leads to Browning of White Adipose Tissue through Type 2 Immune Signaling. Cel Metab. 24, 434–446. 10.1016/j.cmet.2016.07.023 27568549

[B21] Farmer B. C. Walsh A. E. Kluemper J. C. Johnson L. A. (2020). Lipid Droplets in Neurodegenerative Disorders. Front. Neurosci. 14, 742. 10.3389/fnins.2020.00742 32848541 PMC7403481

[B22] Ferrara D. Montecucco F. Dallegri F. Carbone F. (2019). Impact of Different Ectopic Fat Depots on Cardiovascular and Metabolic Diseases. J. Cel Physiol 234, 21630–21641. 10.1002/jcp.28821 31106419

[B23] Gemmink A. Goodpaster B. H. Schrauwen P. Hesselink M. K. C. (2017). Intramyocellular Lipid Droplets and Insulin Sensitivity, the Human Perspective. Biochim. Biophys. Acta (Bba) - Mol. Cel Biol. Lipids 1862, 1242–1249. 10.1016/j.bbalip.2017.07.010 28739280

[B24] Handa R. J. Larco D. O. Wu T. J. (2014). Steroid Hormone Action in Health and Disease: Reference Module in Biomedical Sciences. Amsterdam, Netherlands: Elsevier.

[B25] Hickman S. Izzy S. Sen P. Morsett L. El Khoury J. (2018). Microglia in Neurodegeneration. Nat. Neurosci. 21, 1359–1369. 10.1038/s41593-018-0242-x 30258234 PMC6817969

[B26] Hwangbo D.-S. Lee H.-Y. Abozaid L. S. Min K.-J. (2020). Mechanisms of Lifespan Regulation by Calorie Restriction and Intermittent Fasting in Model Organisms. Nutrients 12, 1194. 10.3390/nu12041194 PMC7230387 32344591

[B27] Hwangbo N. Zhang X. Raftery D. Gu H. Hu S.-C. Montine T. J. (2021). A Metabolomic Aging Clock Using Human Cerebrospinal Fluid. J. Gerontol. A. Biol. Sci. Med. Sci. 10.1093/gerona/glab212 PMC8974344 34382643

[B28] Johnson A. A. Stolzing A. (2019). The Role of Lipid Metabolism in Aging, Lifespan Regulation, and Age‐related Disease. Aging Cell 18, e13048. 10.1111/acel.13048 31560163 PMC6826135

[B29] Jordan S. Tung N. Casanova-Acebes M. Chang C. Cantoni C. Zhang D. (2019). Dietary Intake Regulates the Circulating Inflammatory Monocyte Pool. Cell 178, 1102–1114.e17. 10.1016/j.cell.2019.07.050 31442403 PMC7357241

[B30] Jové M. Maté I. Naudí A. Mota-Martorell N. Portero-Otín M. De la Fuente M. (2016). Human Aging Is a Metabolome-Related Matter of Gender. Gerona 71, 578–585. 10.1093/gerona/glv074 26019184

[B31] Jové M. Mota-Martorell N. Torres P. Portero-Otin M. Ferrer I. Pamplona R. (2021). New Insights into Human Prefrontal Cortex Aging with a Lipidomics Approach. Expert Rev. Proteomics 18, 333–344. 10.1080/14789450.2021.1940142 34098823

[B32] Kaneko H. Itoh H. Kiriyama H. Kamon T. Fujiu K. Morita K. (2021). Lipid Profile and Subsequent Cardiovascular Disease Among Young Adults Aged. Am. J. Cardiol. 142, 59–65. 10.1016/j.amjcard.2020.11.038 33301771

[B33] Kao Y.-C. Ho P.-C. Tu Y.-K. Jou I.-M. Tsai K.-J. (2020). Lipids and Alzheimer's Disease. Int. J. Mol. Sci. 21, 1505. 10.3390/ijms21041505 PMC7073164 32098382

[B34] Kolovou G. D. Bilianou H. G. (2008). Influence of Aging and Menopause on Lipids and Lipoproteins in Women. Angiology 59, 54S–57S. 10.1177/0003319708319645 18515273

[B35] Lemoine M. (2020). Defining Aging. Biol. Philos. 35, 1–30. 10.1007/s10539-020-09765-z

[B36] Lessig J. Fuchs B. (2009). Plasmalogens in Biological Systems: Their Role in Oxidative Processes in Biological Membranes, Their Contribution to Pathological Processes and Aging and Plasmalogen Analysis. Curr. Med. Chem. 16, 2021–2041. 10.2174/092986709788682164 19519379

[B37] Li J. Wang X. Zhang T. Wang C. Huang Z. Luo X. (2015). A Review on Phospholipids and Their Main Applications in Drug Delivery Systems. Asian J. Pharm. Sci. 10, 81–98. 10.1016/j.ajps.2014.09.004

[B38] Liao C.-Y. Rikke B. A. Johnson T. E. Diaz V. Nelson J. F. (2010). Genetic Variation in the Murine Lifespan Response to Dietary Restriction: from Life Extension to Life Shortening. Aging Cell 9, 92–95. 10.1111/j.1474-9726.2009.00533.x 19878144 PMC3476836

[B39] Lichtenstein A. H. (2013). Fats and Oils, Encyclopedia of Human Nutrition. Cambridge: Academic Press, Elsevier, 201–208.

[B40] Liu B. Page A. J. Hutchison A. T. Wittert G. A. Heilbronn L. K. (2019). Intermittent Fasting Increases Energy Expenditure and Promotes Adipose Tissue browning in Mice. Nutrition 66, 38–43. 10.1016/j.nut.2019.03.015 31207437

[B41] Marschallinger J. Iram T. Zardeneta M. Lee S. E. Lehallier B. Haney M. S. (2020). Lipid-droplet-accumulating Microglia Represent a Dysfunctional and Proinflammatory State in the Aging Brain. Nat. Neurosci. 23, 194–208. 10.1038/s41593-019-0566-1 31959936 PMC7595134

[B42] Martin M. Dotti C. G. Ledesma M. D. (2010). Brain Cholesterol in normal and Pathological Aging. Biochim. Biophys. Acta (Bba) - Mol. Cel Biol. Lipids 1801, 934–944. 10.1016/j.bbalip.2010.03.011 20359547

[B43] McGurk K. A. Williams S. G. Guo H. Watkins H. Farrall M. Cordell H. J. (2021). Heritability and Family-Based GWAS Analyses of the N-Acyl Ethanolamine and Ceramide Plasma Lipidome. Hum. Mol. Genet. 30, 500–513. 10.1093/hmg/ddab002 33437986 PMC8101358

[B44] Meng H. Zhu L. Kord-Varkaneh H. O Santos H. Tinsley G. M. Fu P. (2020). Effects of Intermittent Fasting and Energy-Restricted Diets on Lipid Profile: A Systematic Review and Meta-Analysis. Nutrition 77, 110801. 10.1016/j.nut.2020.110801 32428841

[B45] Mesa-Herrera F. Taoro-González L. Valdés-Baizabal C. Diaz M. Marín R. (2019). Lipid and Lipid Raft Alteration in Aging and Neurodegenerative Diseases: A Window for the Development of New Biomarkers. Int. J. Mol. Sci. 20, 3810. 10.3390/ijms20153810 PMC6696273 31382686

[B46] Mortimer C. E. Müller U. (2003). Chemie. Stuttgart: Thieme.

[B47] Müller-Eigner A. Sanz-Moreno A. de-Diego I. Venkatasubramani A. V. Langhammer M. Gerlini R. (2021). Dietary Intervention Improves Health Metrics and Life Expectancy of the Genetically Obese DU6 (Titan) Mouse. bioRxiv. 10.1101/2020.05.11.088625 PMC9065075 35505192

[B48] Nam K. N. Mounier A. Wolfe C. M. Fitz N. F. Carter A. Y. Castranio E. L. (2017). Effect of High Fat Diet on Phenotype, Brain Transcriptome and Lipidome in Alzheimer's Model Mice. Sci. Rep. 7, 4307. 10.1038/s41598-017-04412-2 28655926 PMC5487356

[B49] Pak H. H. Haws S. A. Green C. L. Koller M. Lavarias M. T. Richardson N. E. (2021). Fasting Drives the Metabolic, Molecular and Geroprotective Effects of a Calorie-Restricted Diet in Mice. Nat. Metab. 3, 1327–1341. 10.1038/s42255-021-00466-9 34663973 PMC8544824

[B50] Pamplona R. Borras C. Jové M. Pradas I. Ferrer I. Viña J. (2019). Redox Lipidomics to Better Understand Brain Aging and Function. Free Radic. Biol. Med. 144, 310–321. 10.1016/j.freeradbiomed.2019.03.016 30898667

[B51] Park M. A. Chalfant C. (2018). Fatty Acid Metabolism, Molecular Life Sciences. New York, NY: Springer, 387–401. 10.1007/978-1-4614-1531-2_613

[B52] Pienkowska J. Brzeska B. Kaszubowski M. Kozak O. Jankowska A. Szurowska E. (2019). MRI Assessment of Ectopic Fat Accumulation in Pancreas, Liver and Skeletal Muscle in Patients with Obesity, Overweight and normal BMI in Correlation with the Presence of central Obesity and Metabolic Syndrome. Diabetes Metab. Syndr. Obes. 12, 623–636. 10.2147/DMSO.S194690 31118724 PMC6506015

[B53] Pifferi F. Laurent B. Plourde M. (2021). Lipid Transport and Metabolism at the Blood-Brain Interface: Implications in Health and Disease. Front. Physiol. 12, 645646. 10.3389/fphys.2021.645646 33868013 PMC8044814

[B54] Poljak A. Nady B. Matthew Wai Kin W. Liu Y. Housseini M. Perminder Singh S. (2020). Lipids, Brain Ageing, Dementia, and Lipidomics, Diagnosis and Management in Dementia. Amsterdam, Netherlands: Elsevier, 183–205.

[B55] Rausch J. Gillespie S. Orchard T. Tan A. McDaniel J. C. (2021). Systematic Review of marine-derived omega-3 Fatty Acid Supplementation Effects on Leptin, Adiponectin, and the Leptin-To-Adiponectin Ratio. Nutr. Res. 85, 135–152. 10.1016/j.nutres.2020.11.002 33482602

[B56] Schulz H. (2013). in Fatty Acid Oxidation, Encyclopedia of Biological Chemistry. Editors Lennarz W. J. Lane M. D. (Burlington: Academic Press, Elsevier), 281–284. 10.1016/b978-0-12-378630-2.00071-2

[B57] Slade E. Irvin M. R. Xie K. Arnett D. K. Claas S. A. Kind T. (2021). Age and Sex Are Associated with the Plasma Lipidome: Findings from the GOLDN Study. Lipids Health Dis. 20, 30. 10.1186/s12944-021-01456-2 33812378 PMC8019182

[B58] Surma M. A. Gerl M. J. Herzog R. Helppi J. Simons K. Klose C. (2021). Mouse Lipidomics Reveals Inherent Flexibility of a Mammalian Lipidome. Sci. Rep. 11, 19364. 10.1038/s41598-021-98702-5 34588529 PMC8481471

[B59] Tu J. Yin Y. Xu M. Wang R. Zhu Z.-J. (2017). Absolute Quantitative Lipidomics Reveals Lipidome-wide Alterations in Aging Brain. Metabolomics 14, 5. 10.1007/s11306-017-1304-x 30830317

[B71] Vinayavekhin N. Homan E. A. Saghatelian A. (2010). Exploring Disease Through Metabolomics. ACS Chem. Biol. 5, 91–103. 10.1021/cb900271r 20020774

[B60] Wadhera R. K. Steen D. L. Khan I. Giugliano R. P. Foody J. M. (2016). A Review of Low-Density Lipoprotein Cholesterol, Treatment Strategies, and its Impact on Cardiovascular Disease Morbidity and Mortality. J. Clin. Lipidol. 10, 472–489. 10.1016/j.jacl.2015.11.010 27206934

[B61] Wang M. Yan Y. Zhang Z. Yao X. Duan X. Jiang Z. (2021). Programmed PPAR-α Downregulation Induces Inflammaging by Suppressing Fatty Acid Catabolism in Monocytes. iScience 24, 102766. 10.1016/j.isci.2021.102766 34286232 PMC8273418

[B62] Welte M. A. Gould A. P. (2017). Lipid Droplet Functions beyond Energy Storage. Biochim. Biophys. Acta (Bba) - Mol. Cel Biol. Lipids 1862, 1260–1272. 10.1016/j.bbalip.2017.07.006 PMC5595650 28735096

[B63] WHO (2020). The Top 10 Causes of Death. Available at: https://www.who.int/news-room/fact-sheets/detail/the-top-10-causes-of-death (Accessed October 21, 2021).

[B64] Wong M. W. Thalamuthu A. Braidy N. Mather K. A. Liu Y. Ciobanu L. (2020). Genetic and Environmental Determinants of Variation in the Plasma Lipidome of Older Australian Twins. Elife 9, e58954. 10.7554/eLife.58954 32697195 PMC7394543

[B65] Wymann M. P. Schneiter R. (2008). Lipid Signalling in Disease. Nat. Rev. Mol. Cel Biol 9, 162–176. 10.1038/nrm2335 18216772

[B66] Yan Y. Wang H. Hu M. Jiang L. Wang Y. Liu P. (2017). HDAC6 Suppresses Age-dependent Ectopic Fat Accumulation by Maintaining the Proteostasis of PLIN2 in Drosophila. Develop. Cel 43, 99–111.e5. 10.1016/j.devcel.2017.09.001 28966044

[B67] Yan Y. Wang H. Wei C. Xiang Y. Liang X. Phang C.-W. (2019). HDAC6 Regulates Lipid Droplet Turnover in Response to Nutrient Deprivation via P62-Mediated Selective Autophagy. J. Genet. Genomics 46, 221–229. 10.1016/j.jgg.2019.03.008 31078436

[B68] Zamboni M. Nori N. Brunelli A. Zoico E. (2021). How Does Adipose Tissue Contribute to Inflammageing? Exp. Gerontol. 143, 111162. 10.1016/j.exger.2020.111162 33253807

[B69] Zanco B. Mirth C. K. Sgrò C. M. Piper M. D. (2021). A Dietary Sterol Trade-Off Determines Lifespan Responses to Dietary Restriction in *Drosophila melanogaster* Females. Elife 10, e62335. 10.7554/eLife.62335 33494859 PMC7837700

[B70] Zubrzycki A. Cierpka-Kmiec K. Kmiec Z. Wronska A. (2018). The Role of Low-Calorie Diets and Intermittent Fasting in the Treatment of Obesity and Type-2 Diabetes. J. Physiol. Pharmacol. 69, 663. 10.26402/jpp.2018.5.02 30683819

